# Antimicrobial peptide gramicidin S is accumulated in granules of producer cells for storage of bacterial phosphagens

**DOI:** 10.1038/srep44324

**Published:** 2017-03-15

**Authors:** Marina Berditsch, Mareike Trapp, Sergii Afonin, Christian Weber, Julia Misiewicz, Joana Turkson, Anne S. Ulrich

**Affiliations:** 1 Karlsruhe Institute of Technology (KIT), Institute of Organic Chemistry, Fritz-Haber-Weg 6, 76131 Karlsruhe, Germany; 2 KIT, Institute of Biological Interfaces (IBG-2), P.O.B. 3640, 76021 Karlsruhe, Germany

## Abstract

Many antimicrobial peptides are synthesized non-ribosomally in bacteria, but little is known about their subcellular route of biosynthesis, their mode of intracellular accumulation, or their role in the physiology of the producer cells. Here, we present a comprehensive view on the biosynthesis of gramicidin S (GS) in *Aneurinibacillus migulanus*, having observed a peripheral membrane localization of its synthetases. The peptide gets accumulated in nano-globules, which mature by fusion into larger granules and end up within vacuolar structures. These granules serve as energy storage devices, as they contain GS molecules that are non-covalently attached to alkyl phosphates and protect them from dephosphorylation and premature release of energy. This finding of a fundamentally new type of high-energy phosphate storage mechanism can explain the curious role of GS biosynthesis in the physiology of the bacterial producer cells. The unknown role of the GrsT protein, which is part of the non-ribosomal GS synthetase operon, can thus be assumed to be responsible for the biosynthesis of alkyl phosphates. GS binding to alkyl phosphates may suggest its general affinity to phosphagens such as ATP and GTP, which can represent the important intracellular targets in pathogenic bacteria.

The cyclic gramicidin S (GS) is a prototypical peptide antibiotic, isolated from soil bacilli in the early 1940s^1^,^2^. It contains two repeats of five amino acids, including the unusual ornithine and ^*D*^phenylalanine (_cyclo_[^*D*^Phe-Pro-Val-Orn-Leu]_2_). GS was one of the first natural compounds for which template-based non-ribosomal peptide synthesis was described^3^, but the localization of the synthetase is still unknown. Despite its unprecedentedly high accumulation in cells (up to 30% of dry cell weight), the cationic GS^2+^ molecule is only moderately soluble in water. It has an amphiphilic structure with a high affinity for cellular membranes. This characteristic feature is the underlying reason for its action as an antimicrobial agent, as GS can not only physically destroy the barrier function of the lipid bilayer^4^,^5^, but it can also interfere with and displace membrane-bound proteins^6^,^7^. These deleterious effects, however, raise the intriguing question how the producing bacteria can prevent themselves from getting damaged. *Aneurinibacillus migulanus* (former *Bacillus brevis*) is indeed resistant to the addition of external GS^8^.

An early study of the subcellular morphology of GS-producing cells did not find any GS-containing intracellular structures in connection with its accumulation^9^. It was suggested that the walls of GS-producing cells, which carry acidic S-layer proteins, can accumulate GS and thereby neutralize the harmful effect of the peptide on the cells’ own respiratory membrane-bound proteins^10^,^11^. Once the organization of the GS biosynthesis operon was established, which contains three structural genes (*grsA, grsB, grsT*)^12^,^13^, the role of the GrsA and GrsB proteins was described as a GS-synthesizing multi-enzyme complex^14^,^15^. The GrsT protein shares homology with fatty acid thioesterases, but its function remains unknown^12^,^16^. In a series of experiments where the fermentation medium was supplemented with specific amino acids, the activity of the GS synthetases was reported to show a paradoxical response to 1% arginine^17^. This amino acid markedly enhanced the yield of GS, but inhibited the synthetase activity in the soluble fraction of the cells. At the same time, the enzymatic activity of the membrane fraction increased more than two-fold, hence a membrane attachment of the GS synthetase was proposed.

Gramicidin S is assumed to play a role in the timing of *A. migulanus* spore outgrowth, and in their heat- and UV-resistance^18^,^19^. Based on the observation that the spores of GS-producing cells, but not of GS-negative mutants, are hydrophobic (as determined by adhesion to hexadecane), GS was suggested to be responsible for preventing their rehydration^20^. Indeed, spore germination can be enhanced by washing the spores (with SDS, ethanol, Ca^2+^), or by incubation at high pH. GS was thus proposed to bind to the external layers of spores to delay germination and outgrowth^21^.

Here, we provide a modified, comprehensive picture to explain how *A. migulanus* produces this unusual membrane-active peptide and why it accumulates high levels thereof. Based on the peripheral membrane localization of its biosynthesis apparatus, the subcellular route of GS production can be described. An unparalleled role for GS in the physiology of *A. migulanus* is proposed, namely the stabilization of phosphagens as an energy resource.

## Results

### Visualization of granules in producer cells

**Figure 1:**
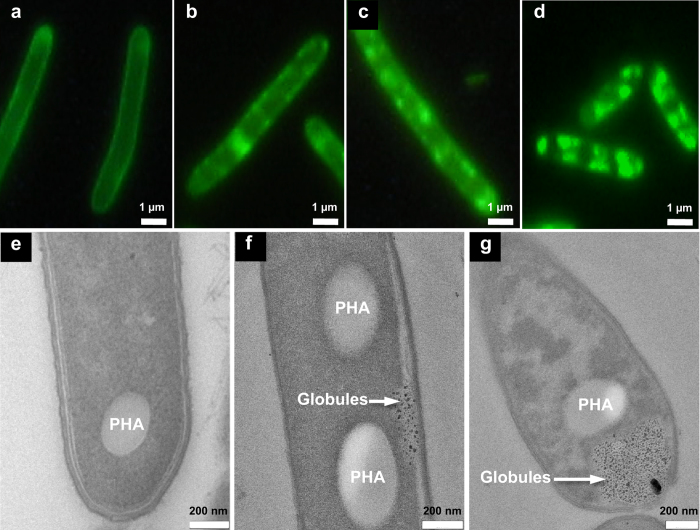
Comparative microscopy of non-producing and GS-producing phenotypes of *A. migulanus* grown in G4/4 medium. (**a**) In the non-producing phenotype, fluorescence is limited to the cell surface. (**b**) In GS-producing cells, at an early stage the granular fluorescence near the cell envelope indicates the presence of GS. (**c**) The fluorescent granular material increases with GS production to a yield of 700 μg/ml peptide. (**d**) Further accumulation of granular structures at a GS yield of 1350 μg/ml. (**e**) Transmission electron microscopy of non-producing cells reveals only large electron transparent granules, i.e. polyhydroxyalkanoates (PHA). (**f**) At the beginning of GS biosynthesis, small electron dense nano-globules appear in the periplasmic space. (**g**) At later stages, a large vacuole at the apical pole gets filled with the electron dense nano-globules.

To visualize the peptide, we compared the rough GS-producing phenotype of *A. migulanus* DSM 5759 with smooth non-producing cells of DSM 5668^8^ after fluorescent staining^22^. Because the dye 5(6)-carboxyfluorescein-*N*-hydroxysuccinimide ester binds to deprotonated NH_2_-groups, at pH 8.3 it can label the weakly basic ornithine side chains of GS molecules. Such staining had been applied earlier to sort GS-hyperproducing cells by means of fluorescence activated cell sorting (FACS)^22^, but FACS does not allow to localize the dye. Hence, we used fluorescence microscopy to directly resolve the stained structures on and within the cells. Since the dye cannot cross the plasma membrane, only GS molecules on the cell surface or those accessible to the dye *via* the periplasmic space should be stained. Indeed, in the non-producing phenotypes, only the cell envelope is stained, as expected, i.e. the S-layer proteins and membrane proteins (Fig. 1a). Notably, the GS-producing phenotypes reveal additional fluorescent granular structures near the membrane (Fig. 1b–d), whose number and size increases with increasing GS accumulation (Fig. 1c,d). These findings suggest an accumulation of GS in the periplasmic space and a subsequent sequestration into these fluorescent, presumably invaginated structures.

Transmission electron microscopy (TEM) of *A. migulianus* DSM 5759 provides better resolution and was used to confirm the presence of the observed granules and to discriminate the complex cell wall structure, consisting of outer S-layer proteins, peptidoglycans, and inner S-layer proteins^9^ (Fig. S1a,d). When the S-layer proteins were removed from GS-producing cells by treatment with acidic glycine buffer, precipitation and washing, a significant amount of GS was always found in the wash buffer. These results corroborate the expected binding of cationic GS to the acidic S-layer proteins^9^. Interestingly, in the TEM images we detected some previously undescribed electron-dense nano-structures that were present only in the GS-producing phenotypes (Fig. 1e–g). These intracellular nano-globules must be related to GS production, as they correlate with the fluorescence data showing small fluorescent particles in tight association with the cell envelope (Fig. 1b,c). During the early stages of GS biosynthesis, the electron-dense structures are located primarily near the plasma membrane in the periplasmic space (Fig. 1f). Further biosynthesis leads to an accumulation of the nano-globules in regions expanding laterally and apically, both in the fluorescent and TEM images (Fig. 1d,g).

**Figure 2:**
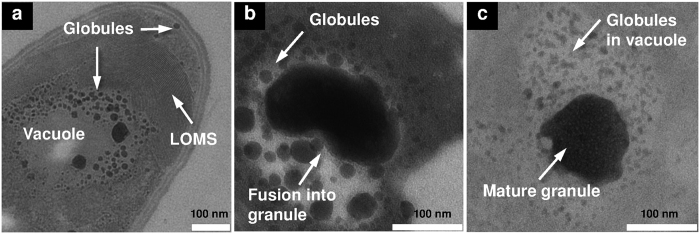
Fusion of nano-globules into granules. (**a**) Lamellar oriented membrane stacks (LOMS) surround the vacuole at the apical pole of cells grown in GATF1 medium for 24 hours. The vacuole is loaded with nano-globules of 5–50 nm diameter. (**b**) Fusion of globules into a large granule continues (GATF1, 48 hours). (**c**) Mature granule consisting of multiple globules (G4/4 medium, 30 hours).

Remarkably, when GS-producing cells were grown in minimal phenylalanine-containing GATF1 medium^23^, they additionally developed lamellar oriented membrane stacks (LOMS, see Fig. 2a), previously unknown for heterotrophic bacteria. Their repeat distance of 4.5 nm is comparable to the thickness of the plasma membrane, and their immediate proximity to the nano-globules suggests a mutual correspondence. The LOMSs are seen to surround a vacuole that is filled with nano-globules (Fig. 2a). At early stages of GS-production, the nano-globules have a typical diameter of 5–50 nm. Over time, however, they fuse into larger round (100 × 100 nm^2^) or ellipsoid (100 × 200 nm^2^) granular structures with the same electron-dense nature (Figs 2b,c and S1c). The estimated sizes of the mature granules in the TEM and scanning electron microscopy (SEM) images are in good agreement (Figs 2b,c and S1c,e). The fusion of nano-globules into larger granules implies a maturation process (Fig. 2b), and it is tempting to speculate that they may contain the newly produced GS.

While the nano-globules are always present in GS-producing cells, the LOMS did not form when cells were grown in the G4/4 medium, which is optimal for GS production^23^. Instead, additional large electron-transparent granules are seen in this medium, resembling the well-known polyhydroxyalkanoate (PHA) granules^24^. These large structures are localized in close proximity to the vacuoles that are filled with the electron-dense nano-globules (Fig. 1g). The occurrence of different subcellular structures (PHA versus LOMS) suggests some variability in the subcellular organization of GS biosynthesis and in the energy management of the cells, depending on nutritional conditions. However, the maturation process of the nano-globules seems to be universal, as their successive enlargement is observed in both growth media (Fig. 2b,c). Interestingly, the mature electron-dense granules in several vegetative cells are observed in close proximity to the segregated DNA filaments. This specific observation suggests that the putative GS-containing granules may be involved in the storage of high-energy phosphates for DNA biosynthesis (Fig. S1a,b). Given the high electron density of these granules, they cannot consist of GS alone but must also contain compounds with a high affinity to the heavy atoms used in staining, i.e. most likely phosphates as in the well-known osmiophilic polyphosphate granules.

### Characterization of isolated granules

Whenever cells had shed their S-layer proteins and cracked, the SEM micrographs showed some granular material outside the cells (Fig. S1d,e). We suggest that this material corresponds to the nano-globules and fused granular structures observed in TEM. It was possible to prepare an aqueous milky suspension consisting of this granular material from the GS-producing cells collected at the end of fermentation, by sonicating the cells and employing differential centrifugation to separate them finally from the large PHA granules (Fig. S2a). In this suspension, the granular material was found to undergo strong aggregation with time (Fig. S2b). The initial average diameter of the particles in the freshly prepared suspension was 130 nm, as estimated by light scattering (Fig. S2c). During overnight storage, it grew to 557 nm (60%) and 175 nm (40%) (Fig. S2d). However, 10 minutes sonication led to disaggregation and reduced the average size of the particles again down to 125 nm (Fig. S2e).

**Figure 3:**
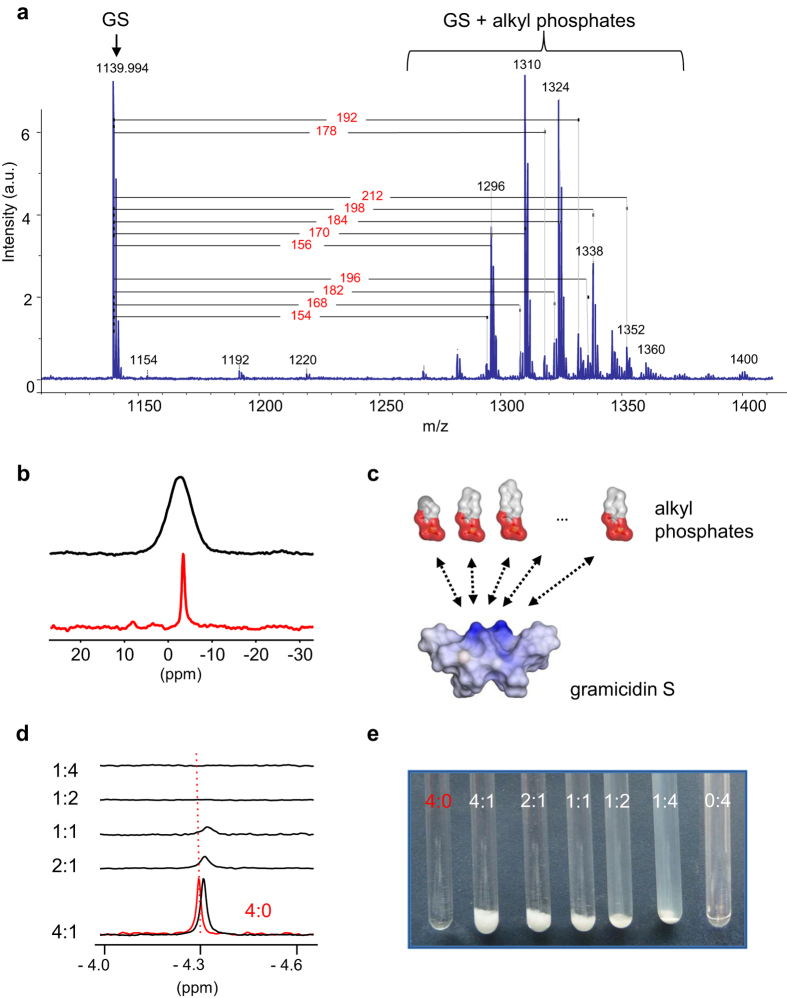
GS binding to alkyl phosphates. (**a**) MALDI mass spectrum of isolated granules revealing the presence of GS ions and associated polymer-like adducts. The mass differences to GS and tentative assignments are shown. (**b**) Solid-state magic angle spinning ^31^P-NMR spectrum of the isolated granules (black trace) and of BuP powder (red trace), under 10 kHz sample rotation at ambient temperature. (**c**) Molecular representation of GS and several alkyl phosphates. The molecules are displayed with their solvent accessible surfaces, coloured according to electrostatics (blue – positive charge, red – negative charge). The molecular dimensions are drawn to scale, and electrostatic interactions are indicated by arrows. (**d**) Liquid-state ^31^P-NMR spectra of BuP:GS mixtures in aqueous suspension (black traces) and of a pure BuP solution (red trace). The millimolar concentrations correspond to the respective numbers given in the ratios. The dashed line corresponds to the reference chemical shift of pure BuP. (**e**) Appearance of the samples that were used to acquire the ^31^P-NMR spectra in panel (d) At elevated GS ratios the formation of an opalescent suspension is observed. The figures are representative of at least two independent experiments in every case.

The accumulation of GS within the intact granules was unambiguously determined by HPLC (data not show), by functional antimicrobial and haemolytic assays of the granular suspension (Table 1), and by mass spectrometry. Interestingly, MALDI spectra of the pelleted suspension (Fig. 3a) always showed – in addition to the GS parent ion (1139.2, [M]^−^) – some regular patterns of ions with higher m/z values, shifted by 154/168/182/196 or 156/170/184/198/212 m/z. In these patterns, the ions are spaced by exactly 14 m/z, which corresponds to the mass of a methylene (CH_2_) segment. A plausible interpretation of these adduct ions may thus be an attachment of GS to a homologous series of alkylated compounds, possessing even and odd numbers of carbon atoms ranging from 3 to 7. Given our earlier speculation that phosphate may be present in the granules, based on their TEM staining behaviour, the first mass ladder could correspond to propionyl/butyryl/pentyl/hexyl phosphates (154/168/182/196 Da), and/or phosphoenol pyruvate (168 Da). The second pattern may represent phosphorylated hydroxyalkanoates, e.g. hydroxybutyryl phosphate with a mass of 184 Da. Even though the assignment is tentative, the FAB spectrum of the granular material after GS extraction confirms the presence of a regular pattern of ions with a predominant mass of 169 Da ([168 + H]^+^) among the other masses (Fig. S3).

The electron-dense granules described in TEM micrographs of various bacteria are typically identified as polyphosphate storage granules^25^. Initially, we therefore hypothesized that the cationic GS^2+^ may interact with such anionic polyphosphates (PolyP), in a similar manner as the divalent cations that are known to stabilize PolyP^26^. To check this possibility, we analysed the isolated GS-containing granules by solid-state magic angle spinning (MAS) ^31^P-NMR to confirm the expected presence of such phosphate chains (Fig. 3b). Surprisingly, only the characteristic signals of terminal phosphate groups are seen (close to 0 ppm), but no PolyP signals could be detected at around −20 ppm^27^. Instead, the simple mono-phosphorylated substrates suggested above based on the MALDI data are found to be fully compatible with the ^31^P-NMR data of the GS-granules. The signals of butyryl phosphate, phosphoenol pyruvate, and AMP are seen between +5 and −5 ppm, whereas PPi and the β-phosphates in ADP and ATP lie between −10 and −25 ppm (Fig. S4). The solid-state NMR data and the MALDI analysis thus corroborate our suggestion that the GS molecules in the granules are coordinated to a family of alkyl mono-phosphates and hydroxyalkyl mono-phosphates with different numbers of methylene units. The high affinity of the anionic phosphates for the cationic GS is obviously driven by electrostatics (Fig. 3c), but must also involve hydrophobic interactions between the alkyl groups and the hydrophobic face of the GS peptide.

To confirm the postulated interaction of GS with butyryl phosphate (BuP) as a representative partner, we studied the response of the liquid-state ^31^P-NMR phosphate signal of an aqueous BuP solution after its addition to dry GS. Mixtures were prepared with BuP and GS in the respective millimolar concentrations that are indicated by the corresponding numbers in each BuP:GS ratio (Fig. 3d,e). BuP on its own is fully water soluble at 4 mM (4:0 sample), but in the presence of 1 mM GS (4:1 sample) the BuP signal gets shifted up-field, indicating an interaction of the phosphate group with the peptide (Fig. 3d). In this sample and the consecutive series with lower BuP:GS ratios, a massive precipitate is seen in the NMR tubes (Fig. 3e). As the ^31^P-NMR signals are found to disappear due to line broadening (Fig. 3d), BuP must be part of the precipitate due to complex formation with GS. Interestingly, in the 1:4 sample, the precipitate becomes almost fully transformed into an opalescent suspension of nano-sized agglomerates, according to SEM data (not shown), which are too large to give an isotropic liquid-state ^31^P-NMR signal.

### Membrane localization of GS synthetases

**Figure 4:**
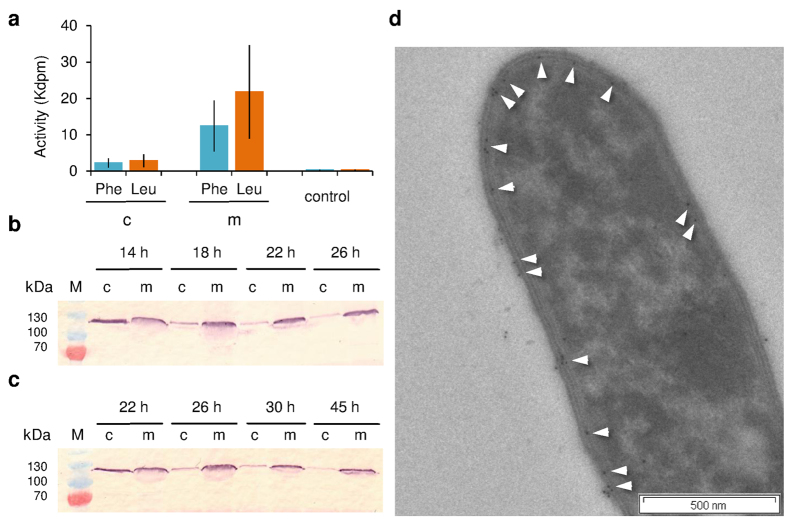
Membrane localization of GS-synthetases in *A. migulanus* DSM 5759 RC phenotype. **(a)** Activity of GrsA and GrsB synthetases in ATP-PPi exchange assays in the membrane (m) and cytoplasmic (**c**) fractions, which were isolated from cultures grown in G4/4 medium. Phenylalanine and leucine were used as substrates for GrsA and GrsB, respectively. Data are means of three independent experiments ± s.d. (**b**) Immunodetection of GrsA in the cytoplasmic and membrane fractions of cells grown in G4/4 medium. (**c)** Immunodetection of GrsA in the cytoplasmic and membrane fractions of cells grown in the minimal GATF1 medium. Data (**b**,**c)** are representative of two independent experiments, each. (**d**) Immunogold staining of GrsA in a GS producing cell from GATF1 medium. White arrows mark the 10 nm gold particles. The figure is representative of at least two independent experiments.

The enzymatic activity of the native GS synthetases was characterized with an ATP-PPi exchange assay (Fig. 4a), using phenylalanine and leucine as substrates for GrsA and GrsB, respectively^28^. The GS-producing strain *A. migulanus* DSM 5759 was cultivated in G4/4 medium to the mid-exponential growth phase, and separated into a membranous and a cytoplasmic fraction. The enzymatic activity of the membrane fraction significantly exceeded that of the cytoplasmic fraction for both substrates (Fig. 4a), and ATP-PPi exchange was generally more pronounced for GrsB (with leucine) than GrsA (with phenylalanine).

For further immunostaining experiments, we obtained polyclonal antibodies against the recombinant phenylalanine adenylation domain (PheA), which comprises the first 600 amino acids of the GrsA synthetase. They were used to monitor the presence of GrsA in the cell fractions of samples collected at different time points by immunostaining. The characteristic 126 kDa band of GrsA was detected at the initial time points of GS production, both in the cytoplasmic and membrane fractions of cells, grown in both G4/4 (Fig. 4b) and GATF1 (Fig. 4c) media. The cytoplasmic signal, however, vanished over time, while the signal in the membrane fraction remained strong throughout. Our observation that the synthetase machinery gets settled on a membrane supports earlier assumptions on its membrane localization^17^. Western blots confirmed that samples incubated with pre-serum did not show any non-specific signals, and no 126 kDa bands were detected in the logarithmic or stationary phases of the non-producing phenotypes (data not shown). Immunogold staining of fixed *A. migulanus* cells also showed independently that GrsA was localized at the plasma membrane, as hardly any gold particles were observed in the cytoplasm (Fig. 4d). In most cases, GrsA was in fact detected in close proximity to the granules described above, i.e. both were at/near the plasma membrane (Fig. S5a) and on/near the membrane of the vacuole (Fig. S5b). These findings clearly demonstrate that the GS biosynthesis apparatus is preferably associated with cellular membranes.

The distinct membrane affinity of the GS synthetases was further confirmed by characterizing the protein-lipid interactions and physico-chemical properties of the proteins involved. A direct lipid-protein overlay assay showed that the recombinant PheA-domain possesses a high affinity for cardiolipin (Fig. S6a), which constitutes up to 25% of the total phospholipid in the plasma membrane of *A. migulanus*^29^. Additional support for the membrane localization comes from a simple hydrophobicity analysis^30^ of the adenylation domains in the GrsA and GrsB synthetases (see Table S1, not shown for GrsB). Namely, five to eight hydrophobic regions were identified in the PheA-domain, depending on the partitioning scale used. When these regions are shown superimposed on the crystal structure of PheA-domain^31^, the protein is seen to be distinctly amphipathic (Fig. S6b). This 3D model provides the structural rationale for postulating a peripheral membrane localization of the enzyme, such that at least one face of the protein is able to interact with the lipid bilayer of the plasma membrane.

## Discussion

The enormous accumulation of GS in the producer cells (up to 30% of dry cell weight) is astounding. Given the high affinity of GS to bind to and permeabilize lipid membranes^5^,^32^, and in view of its dangerous potential to deactivate^6^,^29^ and delocalize^7^ membrane proteins, the question arises as to how GS can be stored without affecting the cells during biosynthesis. Based on its intrinsic affinity for anionic compounds, the binding of GS to S-layer proteins, phospholipids, and/or nucleic acids had been suggested^9^,^33^,^34^, but early TEM studies had not revealed any GS-containing subcellular structures that accumulate GS^9^. Here, we have demonstrated that specific granules containing GS and alkyl phosphates are formed in GS-producing phenotypes of *A. migulianus* to fulfil this task. It had been previously shown that GS biosynthesis is preceded by an elevation of the total lipid content^35^, while the intracellular pools of high-energy nucleotides in the cells are depleted upon GS production^36^. These observations fully support our findings, because extensive phosphorylation has to take place before the fatty acids are stored as alkyl phosphates in granules, in which they are stabilized by complex formation with GS. The tight, non-covalent interaction of GS with these high-energy alkyl phosphates protects them from spontaneous dephosphorylation. This novel concept of protection and storage may have unexpected implications for bacterial physiology in general. For instance, butyryl phosphate is known as a specific substrate in other spore-forming *Firmicutes (Clostridium acetobutylicum)*, where this high-energy molecule - rather than ATP - acts as a direct phosphate donor for transcription factors^37^. The protection of butyryl phosphate and related molecules by binding to GS (or other cationic peptides) could thus provide the producer cells with a mechanism for storing high-energy phosphates in the form of self-assembled peptide-alkyl phosphate complexes instead of the well-known polyphosphate granules. In addition, GS binding to alkyl phosphates suggests its general affinity to intracellular phosphagens (e.g. ATP, GTP), which GS can target in pathogenic bacteria. This affinity serves as additional factor in the previously described antimicrobial and anti-biofilm activity of GS^38^. Remarkably, the haemolytic effect of GS in granules is essentially lower than of GS in solution (Table 1) that could be very promising for the application of GS granular material as an antimicrobial formulation.

The involvement of alkyl phosphates in the formation of storage granules that accumulate GS allows us to speculate on the putative role of the GrsT protein. The *grsT* gene encodes a protein of 256 amino acids with unknown function, which is homologous to fatty acid thioesterases^12^,^13^,^16^. The fact that the transcription initiation site in the GS biosynthesis operon is located upstream of the *grsT* gene^12^ indicates the obligatory participation of the GrsT protein in GS production and/or accumulation. Notably, the active site of thioesterases, containing the catalytic motif GHSMG, is also present in acyltransferases^16^ and lipases^39^. Therefore, as a possible function for GrsT we suggest that this protein may catalyse the hydrolysis of fatty acyl chains from ACP and their subsequent phosphorylation, similar to the reaction of phosphate butyryltransferase (butyryl-CoA + phosphate <=> CoA + butyryl phosphate). In cells grown in GATF1 medium, the formation of LOMS may also be correlated with a lipase activity of GrsT for producing alkyl phosphates from phospholipids.

**Figure 5:**
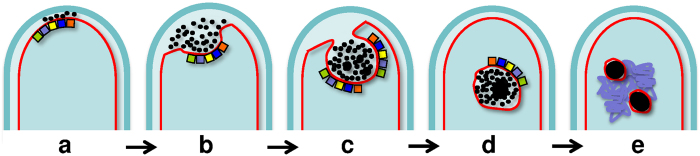
Postulated model for the subcellular organisation of GS biosynthesis in *A. migulanus*. (**a**) At the beginning, both GS synthetases (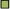
> depicts GrsA; 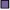, 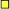, 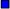 and 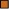 rectangles represent the four modules of GrsB) are peripherally bound to the inner face of the plasma membrane. GS is segregated together with high-energy alkyl phosphates as electron dense nano-globules (black dots) into the periplasm. (**b**,**c**) Upon membrane invagination, the GS-containing nano-globules fill the emerging vacuole. (**d**) Within the vacuole, the small globules fuse into larger GS-containing granules. (**e**) In dormant cells, these granules are located in the region of coiled DNA.

Our discovery of GS-containing granules in connection with the newly established localization of the GS synthetase now permits a fresh view on the subcellular organization of non-ribosomal GS biosynthesis. First, upon binding of the GS synthetase to the inner leaflet of the plasma membrane, GS is accumulated in small nano-globules that are collected in the periplasmic space (Figs 1f and 5a). Subsequently, membrane invaginations (Figs 1g and 5b) lead to the formation of vacuoles as a major repository of the nano-globules, which successively fuse with one another and grow in size (Figs 2 and 5c,d). Finally, in metabolically inactive dormant cells, the vacuoles tighten, and the membrane-enclosed mature granules remain in the vicinity of the coiled DNA (Figs 5e and S1b). They are ready to make their energy-rich phosphates available for DNA synthesis in the next cycle of replication.

These peculiar insights into the subcellular organization of the non-ribosomal peptide synthesis machinery in *A. migulianus* may have practical implications for the engineering of cell-free templated pathways for the production of model peptides, as lipid membranes clearly play a key role in this process. Bacterial granules, containing antimicrobial peptides and high-energy alkyl phosphates, could be utilized in agriculture to enhance the phosphate status of soils, to affect plant pathogens and thereby favourably modulate plant resistance.

## Materials and Methods

### Bacterial strains and growth conditions

*A. migulanus* strains DSM 5759 and DSM 5668, were obtained from the German Collection of Microorganisms and Cell Cultures (DSMZ, Braunschweig, Germany). GS-producing rough convex phenotype (RC of DSM 5759) and non-producing smooth convex phenotype (SC of DSM 5668) were characterized previously^8^ and maintained as spore suspensions in 30% glycerol at −20 °C. To preserve the phenotype characteristics, 1 ml of the amino-nitrogen-rich, yeast-peptone complex medium containing 5 g/l amino-nitrogen^23^ was inoculated with 10 μl of a spore suspension and cultivated at 37 °C without agitation for 48 hours. Then, the cells were plated on modified Luria broth agar medium, containing 10 g/l bacto-tryptone and 10 g/l yeast extract (giving 1 g/l amino-nitrogen), 5 g/l NaCl and 30 g/l agar-agar. At this stage, the colonies were controlled for phenotype stability^23^. Three to five-day-old colonies were used to inoculate chemically defined G4/4 or GATF1 media, described earlier^23^. Fermentation was performed under aeration (agitation at 220 r.p.m.) at 40 °C, until an optical density of 8–10 at 660 nm (OD_660_) was achieved.

The minimum inhibitory concentrations (MIC) of GS were determined *via* a broth microdilution procedure on the control strains^40^: *Escherichia coli* DSM 1103, *Pseudomonas aeruginosa* DSM 1117, *Staphylococcus aureus* DSM 1104, *Enterococcus faecalis* DSM 2570, as well as on *Streptococcus mutans* DSM 20523, which all were purchased from DSMZ.

### GS isolation and quantification

The isolation and quantification of GS was performed as previously described, by extraction from the cells with 50 % acidic ethanol and RP-HPLC^8^,^23^.

### GS visualization by fluorescent staining of cells

At different cultivation times, samples were removed from the culture, diluted to 1 ml with an OD_660_ = 0.3, and centrifuged at 3,500 × *g* at room temperature (RT). The pellet was washed twice and resuspended in fresh 150 mM NaHCO_3_ buffer, pH 8.3. The 5(6)-carboxyfluorescein-*N*-hydroxysuccinimide ester dye was prepared as a 10 mg/ml stock solution in DMSO. For cell staining, 1 μl of the stock solution was added to a 1 ml aliquot of suspended cells. After 30 min incubation at 37 °C with paused agitation, the cells were pelleted, washed twice in 150 mM NaHCO_3_ buffer, and resuspended in 20 μl of the same buffer. The fluorescent structures and GS granules were observed using an Axioskop 40 light microscope (Carl Zeiss Light Microscopy, Göttingen, Germany), equipped with an “A-Plan” objective (100x/1.25 Ph3) anda fluorescence filter (type 09, λ_ex_ 450–490, λ_em_ 515).

### Preparation of samples for electron microscopy

At defined cultivation time points, aliquots were removed and diluted to yield a 1 ml suspension with an OD_660_ of approximately 5. For removal of the S-layer proteins, the biomass was incubated in glycine buffer (200 mM glycine/HCl, 5 mM MgCl_2_, 10 μM glutathione, 250 μM EDTA, pH 2.5) for 2 hours at RT. Unless otherwise stated, 150 mM sodium phosphate buffer (SPB, pH 7.2) was used to prepare the solutions for fixation and for the cell washing procedures. The cells were fixed in 2% glutaraldehyde for 2 hours at RT, followed by an overnight incubation in 0.2% OsO_4_ at 4 °C (for better contrast). Next, the cells were washed twice with SPB and twice with 30% ethanol, followed by staining for 2 hours with 3% uranyl acetate in 30% ethanol. Gradual dehydration was performed using an ethanol-acetone series: 50% ethanol, RT, 10 min; 70% ethanol, 4 °C, overnight; 90% acetone, RT, 10 min; 100% acetone, RT (three times within 2 hours). The samples were embedded in epoxy resin using the Embed-It low-viscosity epoxy kit (Polysciences, Eppelheim, Germany) or in acryl resin LR White (Sigma-Aldrich), which was polymerized en bloc. Ultrathin sections were prepared using a Leica EM UC6 ultramicrotome (Leica Mikrosystem, Wetzlar, Germany) and placed on formvar-coated grids (Plano, Wetzlar, Germany). For immunogold staining, the grids were first blocked in a 2% fat-free BSA solution and incubated overnight with polyclonal PheA antibodies. Next, they were co-incubated with 10 nm sized gold-conjugated anti-rabbit antibodies (Sigma-Aldrich) for 1 hour, and contrasted with 1% uranyl acetate. During immunostaining, SPB containing 0.05% Tween 20 was used. Transmission electron microscopy (TEM) was performed with a Zeiss 912 Omega (Oberkochen, Germany) microscope at 120 keV of electron flow. To optimize contrast, zero-loss energy filtering was applied.

For scanning electron microscopy (SEM), the cells were fixed in 2% glutaraldehyde, washed in SPB, and resuspended in ultrapure MilliQ water. A 1 μl droplet was deposited on a silicon support (Plano, Wetzlar, Germany) and air-dried. Before SEM observation, a thin platinum layer of 1 nm was sputtered onto the sample using a high vacuum coating system Leica EM MED020 (Leica Microsystems, Wetzlar, Germany) to avoid charging by the microscope. The samples were observed with a Zeiss Supra 55VP microscope (Oberkochen, Germany). All reagents applied were of EM grade.

### ATP-PP_i_ exchange assay

Native GS synthetases were isolated from cells grown in the mid-exponential phase, as previously described^12^. The concentrations of the proteins in the cytoplasmic and membrane fractions were estimated using a NanoDrop spectrophotometer (Thermo Scientific, Wilmington, USA). To the 500 μl reaction mixtures, which contained 1 mM ATP, 5 mM DTT, 5 mM NaPP_i_ ·10H_2_O, 10 mM Phe or Leu, and 250 μg/ml enzyme, we added 100 μl of a 50 mM aqueous [γ-^32^P] NaPP_i_ ·10H_2_O solution and incubated for 1 hour. The remaining radioactivity was quenched by the addition of 750 μl of termination solution (100 mM NaPP_i_ ·10H_2_O, 560 mM HClO_4_) supplemented with 1.6% (w/v) charcoal. After washing twice, the pellet was incubated in 750 μl of termination solution. Radioactivity was determined in 15 ml of LumaSafe Plus scintillation cocktail (PerkinElmer, Boston, USA) employing the liquid scintillation analyser Tri-Carb^®^ 2100 (PerkinElmer).

### Production of the PheA protein

The adenylation domain of GrsA synthetase (PheA protein) containing the first 600 amino acids of GrsA was expressed recombinantly in *E. coli* M15 cells using the pQE60 vector. Protein purification was performed *via* Ni^2+^ affinity chromatography, followed by TEV cleavage of the His-tag and size-exclusion chromatography. Purity was checked by SDS-PAGE. The purified protein was used to produce the polyclonal antibody in rabbits (BioCat, Heidelberg, Germany).

### Western blotting

*A. migulanus* cells were cultivated to defined time points and diluted to 1 ml cell suspensions with an OD_660_ = 4. The cells were washed and resuspended in 5 ml of buffer (50 mM Tris-HCl, 20 mM MgCl_2_, 1 mM glutathione, 0.2 mM EDTA, pH 7.4) for disruption *via* sonication on ice. After separation from the cell debris by centrifugation for 15 minutes at 3,000 × *g*, the membrane and cytoplasmic fractions were obtained by centrifugation for 1 hour at 100,000 × *g*. The cytoplasmic proteins were precipitated with 100% acetone overnight at −20 °C. Both fractions were diluted in 60 μl of SDS sample buffer. The proteins were separated by SDS-PAGE and transferred to a Protran BA83 nitrocellulose membrane (Sigma-Aldrich) for Western blotting. Immunodetection was performed with the polyclonal antibodies against PheA and alkaline phosphatase-conjugated anti-rabbit antibodies (Invitrogen, Darmstadt, Germany) according to manufacturer’s instructions. Control blots were performed with the corresponding pre-serum.

### Lipid-protein overlay assay

The lipid-protein overlay assay was performed using the protocol reported in ref. 41. Briefly, the lipids were dissolved in chloroform/methanol (7:3 vol:vol) to yield 16.7 mg/ml solutions. 3 μl of each lipid solution was spotted on a nitrocellulose membrane filter with a pore size 0.45 μm, and dried for 1 hour. Free binding sites were blocked with 3% fat-free BSA. After washing with SPB buffer supplemented with 0.05% Tween 20 and fat-free 3% BSA, the membrane was incubated in the PheA protein solution (10 μg/ml) overnight at 4 °C. Immunodetection was performed as described for the Western blot.

### Hydrophobicity analysis

The hydropathy of the PheA protein was analysed using Membrane Protein Explorer (MPEx 3.2)^30^. Regions in the PheA sequence that were identified as potential membrane-interacting segments simultaneously in the “interfacial” and the “octanol” whole-residue partitioning scales were visualized in the crystal structure of the PheA protein^31^ using Discovery Studio 3.0 software from BIOVIA.

### Isolation and characterization of GS granules

Bacterial cells cultured in G4/4 media were harvested in the stationary phase by centrifugation at 6,000 × *g* (4 °C, 15 min) and washed twice in physiological saline solution. The biomass was concentrated 20-fold in buffer containing 50 mM Tris-HCl (pH 7.2), 1 mM glutathione, 1 mM MgCl_2_/6H_2_O and 0.2 mM EDTA. Then, the cells were sonoporated on ice (twice for 3 min) to release the cell contents. The yield of the granular material was controlled microscopically. The cell debris was pelleted for 10 min at 600 × *g*, and the supernatant was consecutively applied to differential centrifugation at 2,000 × *g*, 5,000 × *g* and 9,000 × *g* (each step for 10 min at 4 °C). The last supernatant was treated with Benzonase^®^ Nuclease (Sigma-Aldrich) for 1 hour at RT and overnight at 4 °C, and then centrifuged at 100,000 × *g* for 30 minutes at 4 °C. The transparent, yellowish pellet was resuspended in sterile ultrapure MilliQ water and lyophilized for storage.

For analysis, this powder of GS granules was dispersed in water to obtain an opaque colloidal liquid. After centrifugation of the granules at 17,000 × *g* for 3 min, the pellet was dissolved in either acidic 50% ethanol (containing 20 mM HCl) or acidic DMSO (containing 0.1% trifluoroacetic acid). HPLC was used to quantify the GS content. The size homogeneity of the granules was examined using a Nano S Zetasizer (Malvern Instruments, UK).

Suspensions of the GS granules were prepared to contain 1 mg/ml of GS in water or 4 mg/ml of GS in isotonic buffer (Tris-HCl 172 mM, pH 7.6 at 37 °C), in order to determine the antimicrobial and haemolytic activities, respectively. For MIC tests, the standard broth microdilution assay was performed in microtiter plates using Mueller Hinton medium as described earlier^38^. MIC was performed with three biological replicates in at least two independent experiments. For haemolytic activity, human blood in citrate-phosphate-dextrose buffer was obtained from the blood transfusion department of the municipal hospital in Karlsruhe, washed twice in isotonic Tris-HCl wash-buffer (pH 7.6, RT), and diluted to 0.5 or 5% of haematocrit in Tris-HCl reaction-buffer (pH 7.6, 37 °C). GS was prepared as an aqueous solution with a concentration of 800 μg/ml by adding 1/10 volume of DMSO and then 9/10 volume of Tris-HCl reaction-buffer. This GS solution or a suspension of GS granules were diluted 1:1 in Tris-HCl reaction-buffer to an end volume of 200 μl, then 200 μl erythrocyte suspension were added to start the haemolytic reaction (37 °C, paused agitation, 30 minutes). The studied concentrations were in the range of 5–200 μg/ml for the GS solution, and 10–1000 μg/ml for the suspension of GS granules. After centrifugation, the 350 μl supernatant containing the released haemoglobin, was characterized in a spectrophotometer at 540 nm. The haemolytic concentrations (HC_90_) were calculated as the percentage haemolysis compared to 100% induced by 0.1% Triton X-100.

For MALDI-TOF mass spectrometry, an initial aqueous suspension of GS granules was centrifuged (17,000 × *g* for 3 min at RT), and the pellet was redissolved in a small amount of acidic DMSO. 1–2 μl of the resulting sample were co-crystallized with 2,5-dihydroxybenzoic acid (1:2 vol:vol water:acetonitrile, 0.1% TFA). The spectra were collected on a Bruker Autoflex III instrument, employing a negative ion acquisition mode and a reflectron in the spectral window of 800–4000 Th.

For FAB mass spectrometry, the pellet of GS granules was treated with acidic 50% ethanol, containing 20 mM HCl, heated at 80 °C for 15 minutes, and then agitated for 1 hour at 30 °C. After centrifugation the pellet was dissolved in glycerol and measured in negative mode using FAB spectrometer MAT95 (MasCom, Bremen, Germany).

### ^31^P-NMR spectroscopy

The pellet of GS granules was washed twice in MilliQ water and dried at ambient temperature. A powder of the GS granules and the butyryl phosphate di-lithium salt (Toronto Research Chemicals, North York, Canada) were each packed into a 4 mm ceramic rotor (Bruker) and measured at RT using a Bruker HXY 4 mm MAS probe on a Bruker AVANCE 500 MHz spectrometer equipped with a wide-bore magnet. Solid-state magic angle spinning (MAS) ^31^P-NMR spectra were collected at variable spinning rates (2–16 kHz) at a 202.4 MHz operating frequency, employing a single-pulse (2.9 μs) experiment, with continuous-wave ^1^H decoupling. At least 16 scans were accumulated for each spectrum, with an inter-pulse delay of 5 s. The spectra were processed with TopSpin 3.1 software.

To determine the binding affinity of GS to butyryl phosphate, GS aliquots, calculated for 1, 2 and 4 mM concentrations in 600 μl, were lyophilized in 2 ml glass vessels. A freshly prepared 600 μl of 4 mM, 2 mM and 1 mM butyryl phosphate (BuP) solutions in D_2_O (pD 7.2) were added to the appropriate amount of GS, in order to create a range of molecular ratios of BuP:GS of 4:1, 2:1, 1:1, 1:2 and 1:4. A sample of pure 4 mM GS was also prepared to demonstrate the low solubility of this peptide in Fig. 4e. Each NMR sample was incubated at RT for at least 2 hours to allow any pellet to precipitate. Each measured reference compound (0.2 mM) was prepared in MilliQ water mixed with 10% D_2_O (Fig. S4). Proton-decoupled ^31^P-NMR spectra were acquired on a Bruker AVANCE 400 MHz spectrometer operating at a ^31^P frequency of 161.9 MHz. The experiments were performed at RT using a Bruker 5 mm BB-PABBO probe. A single-pulse (15 μs) experiment was performed. For ^1^H decoupling, a waltz16 decoupling sequence was used. 3500 scans were accumulated for each spectrum, with an inter-pulse delay of 2 s. The spectra were processed with TopSpin 3.1 software.

## Additional Information

**How to cite this article**: Berditsch, M. *et al*. Antimicrobial peptide gramicidin S is accumulated in granules of producer cells for storage of bacterial phosphagens. *Sci. Rep.* **7**, 44324; doi: 10.1038/srep44324 (2017).

**Publisher's note:** Springer Nature remains neutral with regard to jurisdictional claims in published maps and institutional affiliations.

## Supplementary Material

Supplementary Information

## Figures and Tables

**Table 1:** Biological activity of GS granules.

Sample	MIC (μg/ml) for bacteria					HC_90_ (μg/ml) for haematocrit	
	*E. coli*	*P. aeruginosa*	*S. aureus*	*E. faecalis*	*S. mutans*	0.25%	2.5%
GS solution	16	32	4	8	2	12 ± 3	28 ± 4
GS granules	16	64	4	8	2	38 ± 2	676 ± 48

MIC was performed with three biological replicates in at least two independent experiments. HC_90_ data are means of three independent experiments ± s.d.
