# Supplementary material for: Antimicrobial peptide gramicidin S is accumulated in granules of producer cells for storage of bacterial phosphagens
Source: Sci Rep. 2017 Mar 15;7:44324. doi: 10.1038/srep44324 (PMC5353757; doi:10.1038/srep44324)
Supplement: Supplementary Information [file srep44324-s1.pdf]

Supplementary Information

Antimicrobial peptide gramicidin S is accumulated in granules of producer cells for storage of bacterial phosphagens

Marina Berditsch[1], Mareike Trapp[1], Sergii Afonin[2], Christian Weber[1], Julia Misiewicz[1], Joana Turkson[1] and Anne S. Ulrich[1, 2*]

[1]*Karlsruhe Institute of Technology (KIT), Institute of Organic Chemistry, Fritz-Haber-Weg 6, 76131 Karlsruhe, Germany;*

[2] *KIT, Institute of Biological Interfaces (IBG-2), P.O.B. 3640, 76021 Karlsruhe, Germany*

* Correspondence and requests for materials should be addressed to A.U. (Anne.Ulrich@kit.edu).

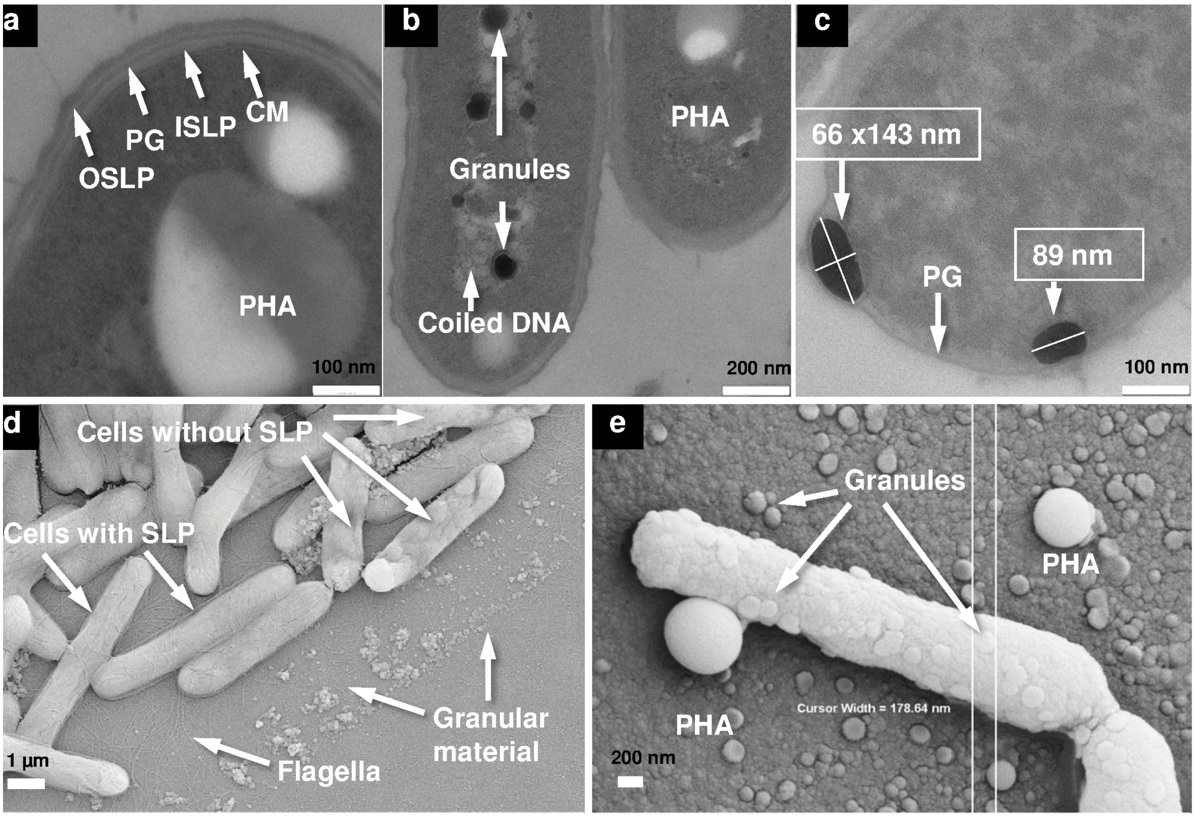

Figure S1. Subcellular morphology of *A. migulanus*. (**a**) Cell wall consisting of outer S-layer protein (OSLP), peptidoglycan (PG), inner S-layer protein (ISLP) and cell membrane (CM). (**b**) Localization of GS containing granules in the region of coiled DNA in metabolically inactive cells. (**c**) Size estimation of the granules. They are seen to move to the cell periphery after glycine buffer treatment of the cells. When the S-layer proteins are washed off, only the peptidoglycan layer is observed in the cell wall. (**d**) Cells that are covered with S-layer proteins are found to be intact in the SEM micrograph, whereas those cells that have shed their S-layer proteins are seen to be disrupted and have release their granules into the environment. (**e**) Two types of granules are distributed over the silicon support when the S-layer protein was washed off and the neighbouring cells got ruptured.

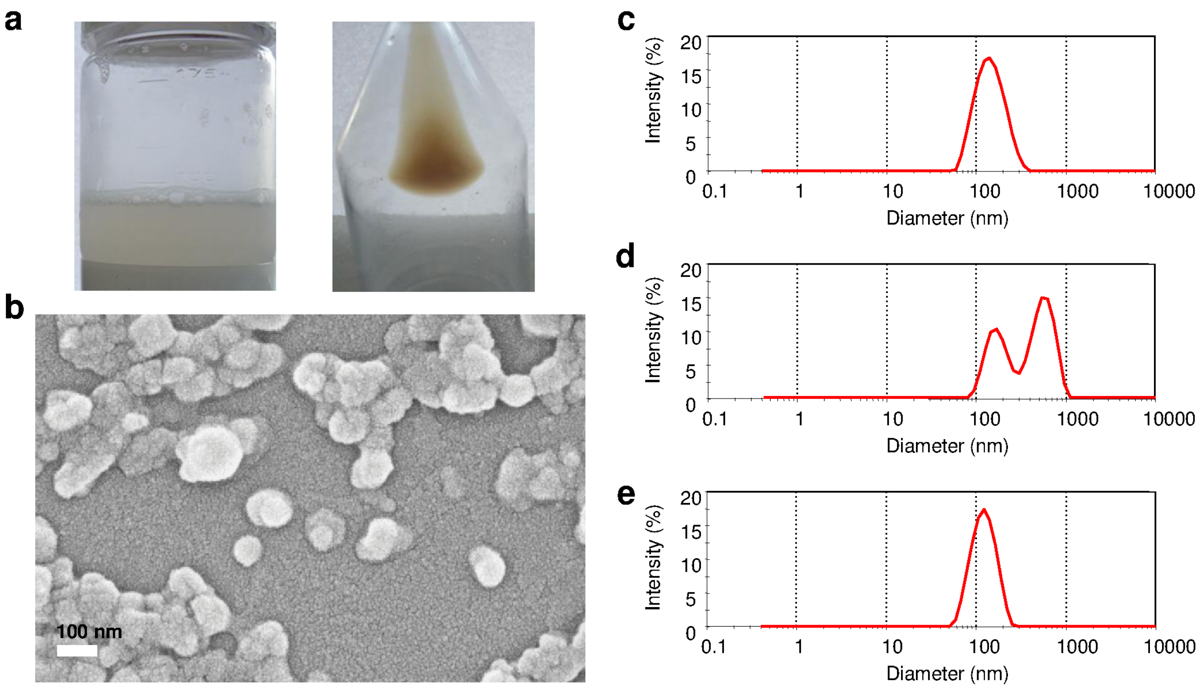

Figure S2. Isolation and characterization of fluorescent electron-dense GS granules. (**a**) Opaque suspension of the granules (left), and solid pellet (right, upside-down tubes) obtained after centrifugation at 9,000×*g*. (**b**) SEM micrograph of the purified granules reveals some agglomeration. (**c**) The size distribution of the granules in a freshly prepared suspension ranges from 50 to 400 nm, with an average particle size of 130 nm. (**d**) After storage, the particle size increases due to agglomeration of the granules. (**e**) Sonication decrease in the average particle size again down to 125 nm. The figures are representative of five independent experiments.

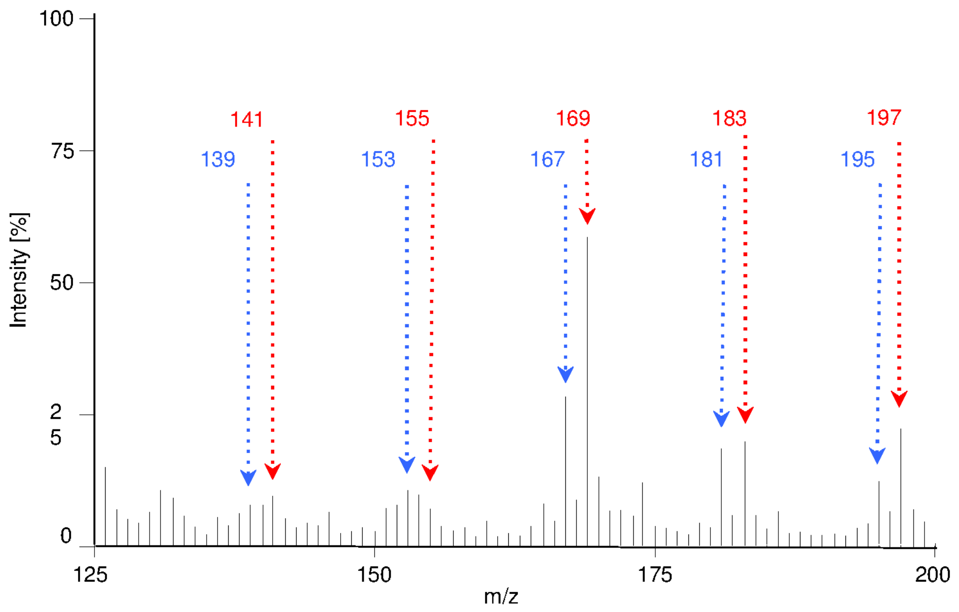

Figure S3. FAB mass spectrometry of the granules remnants after extraction of GS. Regular patterns of ions show the same masses as in the MALDI spectrum, representing the row of alkyl phosphates, where signals belonging to protonated molecules are labelled in red, and deprotonated ones in blue. The figure is representative of two independent experiments.

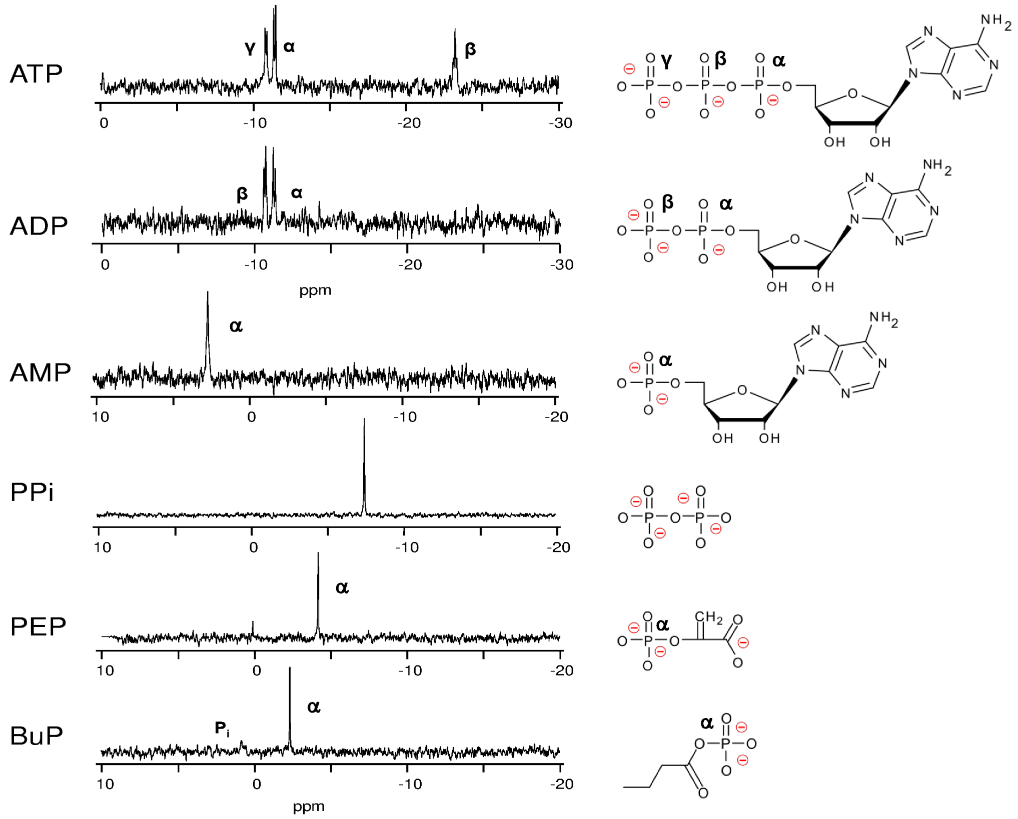

Figure S4. ^{31}P-NMR signals of various phosphorylated compounds. The chemical shifts of the terminal phosphate groups in BuP, phosphoenolpyruvic acid (PEP), PPi as well as the α-phosphate groups in AMP, ADP and ATP vary between +5 and -12 ppm. Only the β-phosphate in ATP exhibits a signal at -23 ppm, which allows a clear discrimination from terminal phosphates.

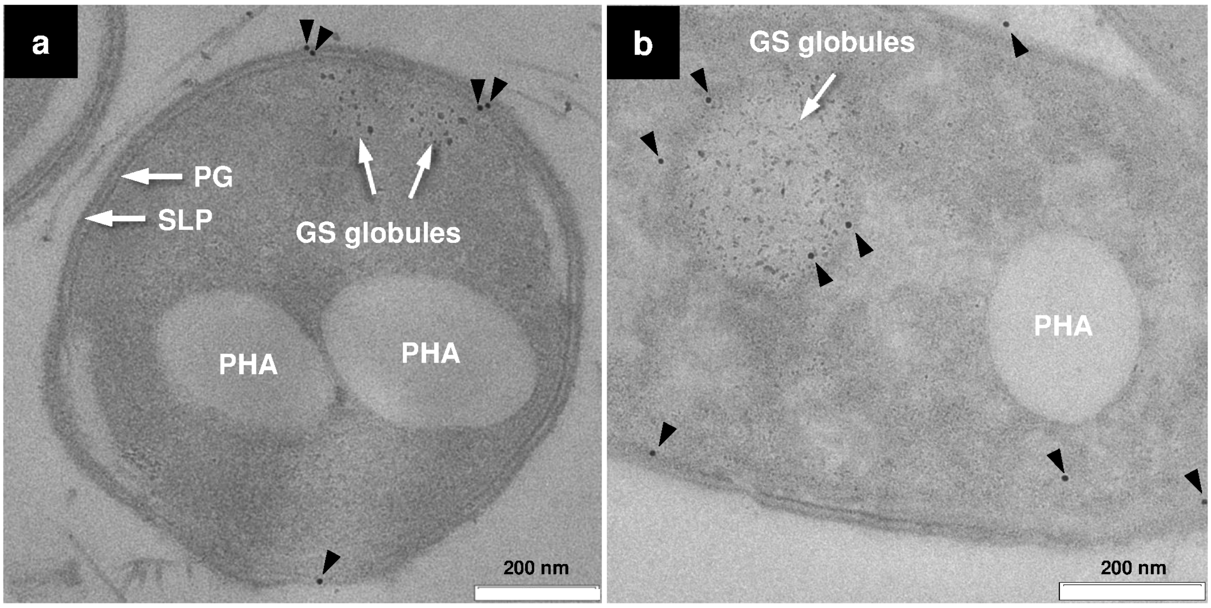

Figure S5. Immunogold TEM imaging of GrsA in GS-producing cells. Cells were grown in G4/4 medium and embedded within epoxy resin. (**a**) GrsA is seen to occur in proximity to the membrane and to the electron-dense GS nano-granules. (**b**) GrsA synthetase is found to reside on the membrane of the vacuole that contains GS nano-globules. In G4/4 medium, a large amount of electron-transparent PHA granules are formed. Black arrows indicate the 10-nm gold particles used to localize GrsA.

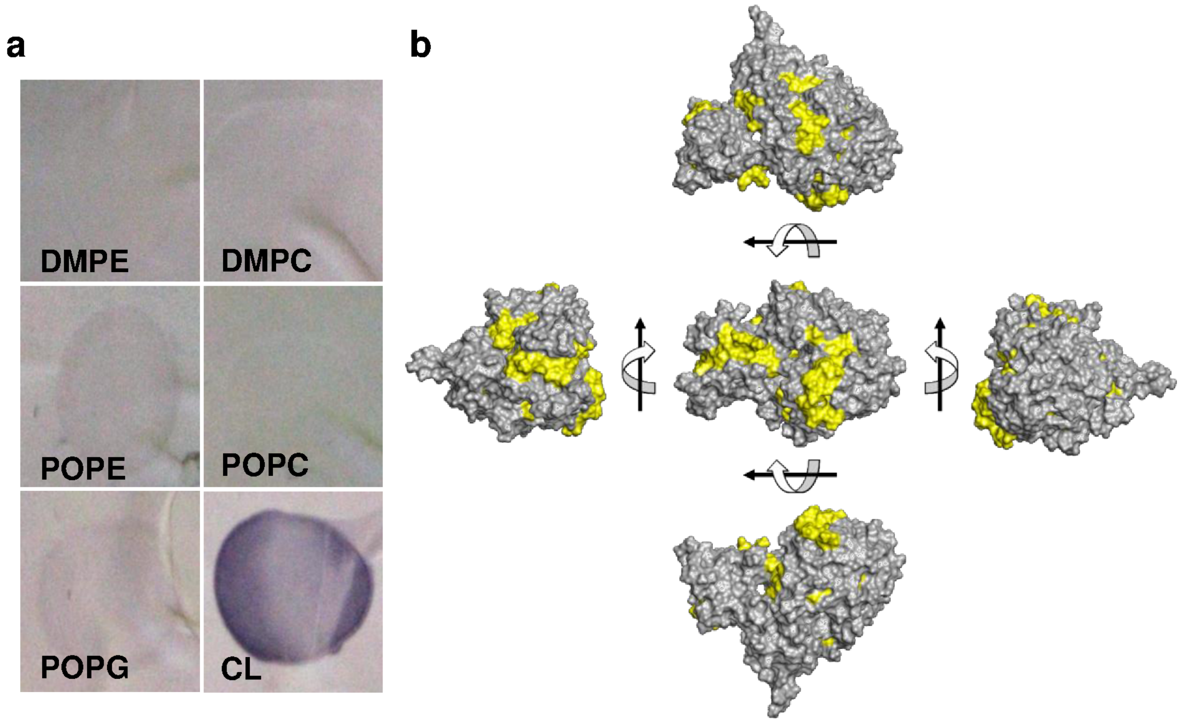

Figure S6. The amphiphilic nature of the PheA-domain suggests a membrane localization of the GrsA synthetase. (**a**) Interaction of recombinant PheA with different phospholipids, showing a high affinity for cardiolipin (CL), as observed in a lipid-protein overlay assay. The figure is representative of two independent experiments. (**b**) Hydrophobic regions of PheA with negative membrane-binding energies (as identified by hydropathy analysis, see Table S1) are displayed on the surface of the 3D model in yellow.

Table S1. Potential membrane-interacting hydrophobic segments of PheA. The respective partitioning energies were determined using the “interfacial” and “octanol” whole-residue partitioning scales [30].

Hydrophobic region	“interfacial” scale	“octanol” scale
AA99-117: SIDLFIGILAVLKAGGAYV	$\Delta G = -1.65$	
AA139-157: MLLTQKHLVHLIHNIQFNG	$\Delta G = -0.50$	$\Delta G = -2.31$
AA184-202: LAYVIYTSGTTGNPKGTML	$\Delta G = -0.72$	
AA237-255: SVWEMFMALLTGASLYIIL	$\Delta G = -4.40$	$\Delta G = -6.60$
AA292-310: ILSIQTLITAGSATSPSLV	$\Delta G = -0.22$	$\Delta G = -1.92$
AA317-335: VTYINAYGPTETTICATTW	$\Delta G = -0.44$	
AA342-360: IGHSVPIGAPIQNTQIYIV		$\Delta G = -0.94$
AA495-513: LPTYMIPSYFIQLDKMPLT	$\Delta G = -1.21$	$\Delta G = -1.87$
